# Indonesian first national suicide prevention strategy: key findings from the qualitative situational analysis

**DOI:** 10.1016/j.lansea.2023.100245

**Published:** 2023-07-04

**Authors:** Sandersan Onie, Ashra Vina, Kezia Taufik, Juneman Abraham, Diana Setiyawati, Erminia Colucci, Jessica F. Nilam, Stephanie Onie, Aliza Hunt, Arif Fajar Saputra, Nurul E. Hidayati, Christine Harsono, Damba Bestari, Nalini Muhdi, Alegra Wolter, Andrian Liem, Ida Rochmawati, Jiemi Ardian, Radityo Eko Prasojo, Yohanes Aristanto Heri Setiawan, Grace Heny, Halim Purnawan, Indria Laksmi Gamayanti, Herwindra Aiko Senosoenoto, Marthen Jenarut, Benny Prawira, Cahyo Trianggoro, Edberg Warbung, Catherine Lily Novita Mudjianto, Anna Surti Ariani, Irmansyah Irmansyah, Musdah Mulia, Jussar Badudu, Maranatha Badudu, Retno Kumolohadi, Rizqy Amelia Zein, Stephanie Mahadi, Turro Wongkaren, Natasha Josifovski, Mark E. Larsen

**Affiliations:** a Black Dog Institute, UNSW Sydney, Australia; b Universitas Airlangga, Surabaya, Indonesia; c Emotional Health for All Foundation, Indonesia; d Indonesian Association for Suicide Prevention, Indonesia; e Bina Nusantara University, Indonesia; f Indonesian Psychological Association, Indonesia; g Centre for Public Mental Health, Universitas Gajah Mada, Indonesia; h Middlesex University, London; i Australia National University, Australia; j University of Sydney, Australia; k Ibunda.id, Indonesia; l Indonesian Association of Social Workers, Indonesia; m Revolver Indonesia, Indonesia; n Perkumpulan Suara Kita, Indonesia; o Monash University Malaysia, Malaysia; p RSUD Wonosari GK-DIY, Indonesia; q Siloam Hospitals, Indonesia; r Pitik Digital Indonesia, Indonesia; s Komisi Keluarga Konferensi Waligereja Indonesia, Indonesia; t 168 Solution, Indonesia; u Yayasan Manusia Indonesia Baru, Indonesia; v Ikatan Psikolog Klinis Indonesia, Indonesia; w Pusat Pengembangan Diri dan Komunitas Kemuning Kembar, Indonesia; x Majelis Nichiren Shoshu Buddha Dharma Indonesia, Indonesia; y JPIC Keuskupan Ruteng, Indonesia; z Into the Light Indonesia, Indonesia; aa Badan Riset dan Inovasi Nasional, Indonesia; ab Tunas Bangsa Christian School, Indonesia; ac Sekolah Lentera Indonesia, Indonesia; ad Religions for Peace Asia, Indonesia; ae Jakarta Praise Community Church, Indonesia; af Universitas Islam Indonesia, Indonesia; ag Open Science Indonesia, Indonesia; ah Bisnis.com, Indonesia; ai Universitas Indonesia, Indonesia

The reduction of suicide is a priority within the United Nations’ Sustainable Development Goals. However, Indonesia—the fourth most populous country globally—does not have a national suicide prevention strategy. Thus, in 2021, we began developing such a strategy, starting with a situational analysis recommended by the WHO LIVE-LIFE framework.^1^ This nationwide effort was led by a leadership committee advised by the Ministry of Health and WHO Indonesia.

During the situational analysis, we investigated risk, protective and unique cultural factors; registry infrastructure; government legislation and processes; healthcare systems and roles; suicide research infrastructure and capacity; current efforts; data needs; and how these factors interact. We applied various methods, including studying non-public historical records, case studies, field interviews and service mapping, applying grounded theory qualitative research methods. The specific methods for the qualitative situational analysis can be found in the appendix. Below we outline context-specific findings, primarily from our qualitative investigations. This report does not cover all findings from the situational analysis and should be read with the Indonesian Suicide Statistics Profile.^2^

While Indonesia does not officially report a national suicide rate, the WHO estimates a low suicide rate (2.6/100,000); however, the WHO has classified the data quality as low.^3^ There are myriad sources of suicide data, including police data, regional administrative surveys, and a death registry, with police data traditionally being accepted as the official source^4^; however no two sources agree, and there have been no efforts to collate and compare raw data from these sources. Further, underreporting is estimated at 859.10%,^2^ currently, the highest national underreporting rate globally.

Our investigation revealed that families often go to extreme lengths to prevent a suicide from being known due to stigma and shame, using any means—including financial–at their disposal so police or doctors do not record a death as a suicide. It was also found that among health professionals, even in the absence of any request, there is an unwritten rule not to mention suicide, a phenomenon we dub *reporting taboo*. Hospitals have no mandate nor standardized pipeline to report suicides. Due to a lack of data quality control, we cannot confidently quantify the true rates of suicide.^2^

Suicide in Indonesia is not criminalised; however, individuals who attempt suicide cannot claim universal insurance (*BPJS*)^5^—on which 225 million individuals are reliant–as suicide is perceived as an individual's free choice to burden society and themselves.^5^ Doctors will remove any indication of suicidal intent to allow patients to access universal insurance—undermining registry-based monitoring.

There is a lack of continuity, coordination, and rigorous adaptation of most suicide prevention efforts. Consistent funding for suicide prevention is sparse, rendering suicide hotlines unsustainable. Research is scant, and reviews of existing local literature are challenging as local journals are not properly indexed in a database. Important context-specific knowledge is either missing or inaccessible. Clinical help is often unreachable, with 4401 psychologists and psychiatrists for the 273 million^6^ population, with very few trained in suicide prevention. Most individuals cannot afford therapy.

Our investigation found that family and religion are key factors that interact in a complex way with suicide. Individuals often cite family issues as a key cause of suicidal intent, but remembering family often prevents an attempt. Similarly, held religious beliefs have both stopped individuals from attempting suicide and stopped individuals from seeking help.

The mention of suicide results in a visceral reaction, leading to an aversion to the issue. Grounded theory analyses revealed that this is likely due to the religion-based moralisation of suicide, given that suicide is considered a deadly sin,^7^ and religion is central to Indonesia's society.

Several action plans are proposed. First, the development and validation of a suicide registry is paramount. Given that priorities outside of suicide largely govern the data stewards’ processes, we propose a new registry that collates and investigates police and hospital records.

Second, given the lack of continuity, coordination, and research in suicide prevention activities, a body responsible for overseeing the implementation and evaluation of these action points and coordinating future efforts is needed. The founding of the Indonesian Association for Suicide Prevention is underway.

Third, given the potential role of religion in suicide stigma, the centrality of religion in Indonesian society, and the influence of religion and religious organizations on national issues, religious organizations can take a central role in suicide prevention in Indonesia. For example, a religious approach to the destigmatisation of the topic of suicide may be warranted, such as a joint statement from religious leaders with theological justifications,^8^ which may permit further efforts to grow, such as lobbying for universal health coverage for suicide attempts and increased research and funding. Local religious leaders can use their platform and forums to foster open discussions about mental health and promote help-seeking. Further, religious organizations are among the best-resourced and coordinated organizations in the country. They can partially support or host key efforts—such as the registry, national association, and local evidence-based research implementation.

Further recommendations include adapted suicide prevention training for clinicians and laypersons, integrating lived experience perspectives into all areas of suicide prevention, and emphasising family and community-based approaches. Given the strong cultural influences noted above, research must focus on understanding Indonesia's unique context and evaluating interventions locally^9^ as there is an imperative to ensure the limited resources available are judiciously used to reduce suicides in the Indonesian community.

## Contributors

The team represents a diverse consortium of researchers, lived experience advisors, and representatives from education, religion, government, media, local non-profit organizations covering mental health, suicide, drug and alcohol rehabilitation, and many more. Sandersan Onie, Ashra Vina, Juneman Abraham, Diana Setiyawati, Erminia Colucci, Jessica F. Nilam, Stephanie Onie, and Kezia Taufik are part of the leadership committee, led by Sandersan Onie and advised by the Indonesian Ministry of Health and World Health Organisation Indonesia. The leadership committee and Mark E. Larsen were involved in the conceptualization, study design and methodology. Mark E. Larsen provided supervisory role. The rest of the authors were interviewed as part of the expert panel and have equal contributorship for data collection. Sandersan Onie and Kezia Taufik were involved in the data collection, formal analysis, and administration. Sandersan Onie wrote the original draft, with the rest of the authors reviewing and editing the manuscript. Apart from the leadership committee and authors with supervisory roles, final authors with the Ministry of Health, names are arranged in chronological order of engagement and are not indicative of contribution.

## Data sharing statement

Aggregate and analysed qualitative data will be shared with key stakeholders who provide a sound rationale. To access the data, correspondence should be directed towards Dr. Sandersan Onie at s.onie@blackdog.org.au or sandy.onie@gmail.com in the case of a defunct institutional email.

## Declaration of interests

SO and KT conducted the interviews for the quantitative study. AH, AFS, NEH, CH, DB, NM, AW, AL, IR, JA, REP, YAHS, GH, HP, ILG, HAS, MJ, BP, CT, EW, CLNM, ASA, II, MM, JB, MB, RK, RAZ, SM, TW were interviewed as part of this study and were given opportunities to revise the manuscript and report it was based on. Apart from these, we declare no competing interests.
