# Supplementary material for: Indonesian first national suicide prevention strategy: key findings from the qualitative situational analysis
Source: Lancet Reg Health Southeast Asia. 2023 Jul 4;16:100245. doi: 10.1016/j.lansea.2023.100245 (PMC10485777; doi:10.1016/j.lansea.2023.100245)
Supplement: Supplementary Methods [file mmc1.docx]

**Methods**

The following methods section is written as a supplementary attachment to the article “Indonesian First National Suicide Prevention Strategy: Key Findings from the Qualitative Situational Analysis” for The Lancet Regional Health: Southeast Asia, to provide more detail on the methodology used.

**Design**

In this study, we conducted semi-structured questionnaires assessing the situation surrounding suicide and issues proximal to suicide, which altogether could be relevant in developing a strategic course of action for the National Suicide Prevention Strategy. This study is not meant to = comprehensively describe suicide prevention on its own, but as a companion to the quantitative data collection method also being undertaken and to cover themes and phenomena that are not immediately quantifiable through the quantitative analysis.

**Topic Selection**

To determine topic selection, our leadership committee for the National Suicide Prevention Strategy, which included the Ministry of Health, WHO Indonesia, local and international subject matter experts, and lived experience advisors, collated a list of relevant topics which was discussed and agreed upon. The focus was to cast as wide of a net as possible to ensure we were capturing all relevant topics, such that our national strategy would enable us to utilise all existing resources and developments. This is in line with existing WHO LIVE LIFE situational analysis guidelines, to ensure that multiple stakeholders would collaborate for suicide prevention.

The topics that were included are: the state of suicide research, lived and living experience of suicide, national and local government policy, research infrastructure for suicide (such as ethics boards, data repositories etc.), existing efforts and their sustainability, healthcare infrastructure (emergency hotlines and their suicide modules), technology (apps and AI), and start-ups relevant to suicide prevention, social work in Indonesia, challenges in the LGBTQI+ community, education and schooling, dynamics in Indonesian families, media, religion.

**Participants**

To capture the wide range of topics above, we conducted 60 interviews, with subject matter experts, including national and international researchers, local and national government, individuals with lived and living experience, advocates from the LGBTQI+ community, non-profit and start-up CEOs, religious leaders, mental health and suicide prevention advocates, the National Research and Innovation Agency, school teachers, family counsellors, and representatives from the Ministry of Health and WHO Indonesia. Each interview lasted on average 1 and a half hours, but we did not limit the time to allow a full understanding of the situation surrounding suicide in Indonesia.

**Materials**

The interview questions were developed by the leadership committee and were designed to not only collect necessary information, but rather prompt meaningful discussion surrounding the nature of suicide in Indonesia. As a result, while ensuring we obtained the necessary information, the interviewee would be encouraged to share other relevant topics. Note that following question 2.1 below, for industries that were not familiar with suicide, a brief 10-minute information presentation was shown to familiarise them with issues surrounding suicide, such that they are able to relate their industry specific expert knowledge to solving this issue.

- Questions Begins -

1. Expert Information

1.1 Name:

1.2 Title:

1.3 Background (Academic, Research Infrastructure and System, Clinician, Government, Policy, NGO, Lived Experience, Education, Technology, DEI Group, other):

1.4 Contact Details:

1.5 Links with Organization:

1.6 (Optional) Do you have lived experience/suicide bereavement?

**2. Suicide, Risk and Protective Factors**

2.1 What do you know about suicide? / Can you tell me one thing about suicide?

2.2 From what you know, what are likely to be the causes and contributing factors of suicide?

2.3 What stops people from dying by suicide?

2.4 Can you talk about stigma towards suicide and people with suicide in Indonesia?

2.5 Why do you think people will not seek help in Indonesia?

**3. Data (only in relevant industries)**

3.1 Do you know the latest suicide statistics in your country?

3.2 Do you know how this information was recorded?

3.3 Other comments:

**4. Government Policy**

4.1 Do you know if suicide currently criminalized?

4.2 Do you know if death certificates are issued and by whom?

4.3 Other comments:

**5. Programs**

5.1 Do you know any suicide prevention programs that are being implemented or planned in the country (for example, it can be by local government, schools, clinics and/or national agencies)?

For example:

Support groups, crisis centers etc.

5.2 From your expertise, what do you think we need or can do to prevent suicide? Also, what can people from your field do to prevent suicide?

5.3 Comments

**6. Culture**

6.1 Can you think of any cultural (traditional, religious, or any) beliefs or practices that influence suicidality?

6.2 Can you think of any unique local cultural practices or incidences that influence suicidality?

**7. Research**

7.1 Are you aware of suicide research projects in Indonesia?

7.2 Can you think of any barriers to good suicide research in Indonesia?

**8. Data collection**

8.1 What data, information, or knowledge would be helpful to tackle suicide in Indonesia?

8.2 If you could ask something to people with lived experience of suicide, what would you ask them?

**9. Services**

9.1 If someone is suicidal, where would they go?

9.2 Do you know what you would do if someone told you they were suicidal?

**10. Other Groups**

10.1 Are you aware of any organizations who touch on suicide and suicide prevention?

- Questions End -

**Data Collection**

A representative from the leadership team contacted each individual identified to be a relevant expert in the subject matter through email and if they responded would schedule a call. Interviews were conducted online over zoom, given that many of these individuals lived in different cities across Indonesia, and that data collection occurred during one of many COVID 19 lockdowns in Indonesia. Every interview had two interviewers who would independently take notes on the discussion.

**Data Preparation**

To prepare the data for analysis, the interviews were transcribed into text with a professional transcription agency to ensure accuracy. The video and text interviews were analysed together, along with notes taken during the interview.

**Data Analysis**

We applied thematic analyses as outlined by Braun and Clarke (2006) with two independent researchers experienced in qualitative analysis for suicide themes. Any findings which we report in the main body of the manuscript were corroborated by at least 3 independent sources. If there were key claims, we would follow up on independent experts to confirm the claim. Throughout the analyses, we a prominent theme of religion and applied grounded theory to further understand how religion pertains to the stigma and aversion of the topic of suicide.
